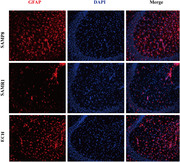# Echinacoside improves the memory impairment of senescence accelerated mouse‐prone 8 (SAMP8) mice via inhibiting glial activation

**DOI:** 10.1002/alz.085863

**Published:** 2025-01-09

**Authors:** Rong Zhou, Ge Tian, Jing Yu, Rui Wang, Xingzhi Guo, Rui Li

**Affiliations:** ^1^ Shaanxi Provincial People’s Hospital, Xi'an, Shaanxi China; ^2^ Shaanxi Provincial People’s Hospital, XI'AN, Shaanxi China; ^3^ Northwest University, XI'AN, Shaanxi China

## Abstract

**Background:**

Cognitive impairment, a common aging‐related pathology, is a risk factor for dementia. Echinacoside (ECH), derived from the traditional Chinese medicine Cistanche deserticola, shows anti‐aging properties including anti‐inflammation, oxidative stress reduction, and neuronal protection. Despite its benefits, the beneficial impact of ECH on age‐related cognitive decline remains unclear. Senescence accelerated mouse‐prone 8 (SAMP8) mice, known for rapid aging and related pathologies in the brain like glial activation, neuro‐inflammation, neuron loss, and cognitive decline, are ideal for this study. The purpose of this study is to investigate the effect of ECH effects on cognitive functions in SAMP8 mice.

**Methods:**

Six‐month‐old male SAMP8 mice (n = 8∼9) were used as the model group, while age‐matched senescence‐accelerated mouse resistant 1 (SAMR1) mice were used as normal controls. After adaptation in the specific pathogen free (SPF) room for one week, we administered ECH intragastrically to the SAMP8 mice daily for two months, and the control group was administered with saline. Behavioral tests, including open field test and Morris water maze, were performed to assess the mood and memory function of the SAMP8 mice. After that, all mice were sacrificed by intraperitoneal perfusion to extract brain tissues for western blotting and immunofluorescence.

**Results:**

ECH‐treated SAMP8 mice showed significantly reduced escape latency in the Morris water maze compared to controls, indicating improved cognitive abilities (P<0.05). ECH also tended to lower beta‐amyloid and phosphorylated Tau levels in the hippocampus of SAMP8 mice, though not statistically significant due to small sample sizes (n = 3). SAMP8 mice had higher microglia and astroglia activation than SAMR1 mice, but ECH notably inhibited this in SAMP8 mice (**Figure 1**).

**Conclusions:**

Our study demonstrates that ECH intervention can markedly enhance the memory function in SAMP8 mice and inhibits microglial and astroglial activation. These findings suggest a beneficial role of ECH in alleviating cognitive decline in SAMP8 mice by reducing glia‐related inflammation.